# Pharmacokinetics and pharmacodynamics of azithromycin in severe malaria bacterial co-infection in African children (TABS-PKPD): a protocol for a Phase II randomised controlled trial

**DOI:** 10.12688/wellcomeopenres.16968.2

**Published:** 2023-01-10

**Authors:** Peter Olupot-Olupot, William Okiror, Hellen Mnjalla, Rita Muhindo, Sophie Uyoga, Ayub Mpoya, Thomas N Williams, Rob terHeine, David M Burger, Britta Urban, Roisin Connon, Elizabeth C George, Diana M Gibb, A Sarah Walker, Kathryn Maitland

**Affiliations:** 1 Mbale Clinical Research Institute, Pallisa Road, PO Box 291, Mbale, Uganda; 2 Busitema University Faculty of Health Sciences, Mbale Regional Referral Hospital, Mbale, Uganda; 3 KEMRI Wellcome Trust Research Programme, PO Box 230, Kilifi, Kenya; 4 Department of Infectious Disease and Institute of Global Health and Innovation, Division of Medicine, Imperial College, London, UK; 5 Department of Pharmacy, Radboud University Medical Center, Radboud Institute for Health Sciences, Nijmegen, The Netherlands; 6 Liverpool School of Tropical Medicine, Pembroke Place, Liverpool, L3 5QA, UK; 7 MRC Clinical Trials Unit, University College London, Aviation House, 125 Kingsway, London, WC28 6NH, UK

**Keywords:** Severe Malaria, Bacterial infection, African Children, Antibiotics, azithromycin, Clinical Trial, Pharmacokinetics, Pharmacodynamics, Biomarkers

## Abstract

**Background: **African children with severe malaria are susceptible to Gram-negative bacterial co-infection, largely non-typhoidal Salmonellae, leading to a substantially higher rates of in-hospital and post-discharge mortality than those without bacteraemia. Current evidence for treating co-infection is lacking, and there is no consensus on the dosage or length of treatment required. We therefore aimed to establish the appropriate dose of oral dispersible azithromycin as an antimicrobial treatment for children with severe malaria and to investigate whether antibiotics can be targeted to those at greatest risk of bacterial co-infection using clinical criteria alone or in combination with rapid diagnostic biomarker tests.

**Methods: **A Phase I/II open-label trial comparing three doses of azithromycin: 10, 15 and 20 mg/kg spanning the lowest to highest mg/kg doses previously demonstrated to be equally effective as parenteral treatment for other salmonellae infection. Children with the highest risk of bacterial infection will receive five days of azithromycin and followed for 90 days. We will generate relevant pharmacokinetic data by sparse sampling during dosing intervals. We will use population pharmacokinetic modelling to determine the optimal azithromycin dose in severe malaria and investigate azithromycin exposure to change in C-reactive protein, a putative marker of sepsis at 72 hours, and microbiological cure (seven-day), alone and as a composite with seven-day survival. We will also evaluate whether a combination of clinical, point-of-care diagnostic tests, and/or biomarkers can accurately identify the sub-group of severe malaria with culture-proven bacteraemia by comparison with a control cohort of children hospitalized with severe malaria at low risk of bacterial co-infection.

**Discussion**: We plan to study azithromycin because of its favourable microbiological spectrum, its inherent antimalarial and immunomodulatory properties and dosing and safety profile. This study will generate new data to inform the design and sample size for definitive Phase III trial evaluation.

**Registration:** ISRCTN49726849 (27 ^th^ October 2017).

## Abbreviations

AQUAMAT: Artesunate versus Quinine in the treatment of severe falciparum Malaria in African children AUC24h: area under the curve at 24 hours; AVPU: alert, voice, pain, unresponsive: system of recording patient’s level of consciousness; CRF: case report form; CRP: C-reactive protein; *E. coli*: *Escherichia coli*; ETAT: emergency triage assessment and treatment; FEAST: Fluid Expansion As a Supportive Therapy; GCP: Good Clinical Practice Hb: haemoglobin; IBI: invasive bacterial infection; ICREC: Imperial College Research Ethics Committee; KWTRP: KEMRI Wellcome Trust Research Programme; MIC: minimum inhibitory concentration MRRH: Mbale Regional Referral Hospital; MRRH-REC: Mbale Regional Referral Hospital Research Ethics Committee; MRC: Medical Research Council; NTS: non-typhoidal salmonella; PCT: procalcitonin; PD: pharmacodynamic; *P. falciparum* HRP2: *Plasmodium falciparum* histidine-rich protein 2; PK: pharmacokinetic; PKPD: pharmacokinetic-pharmacodynamic; RDT: rapid diagnostic test; TRACT: TRansfusion and Treatment strategies of severe Anaemia in African children Trial; WHO: World Health Organization.

## Introduction

*Plasmodium faliciparum* malaria remains to be a common cause of hospital admission in much of sub-Saharan Africa, and plays a substantial role in under five-year mortality ^1^. Strategies to control or prevent malaria (including vaccines) have so far offered limited short-term protection. Over the past decade some African countries have either documented no decline ^2^, an increase in hospitalisations with severe *P. falciparum* malaria ^3^ or a resurgence of severe malaria following a period of sustained control. Thus, prevention strategies alone in many parts of Africa will not adequately address the burden that malaria poses on health services, particularly in countries where transmission is high. Even with the best (evidence-based) antimalarial treatment artesunate, children in the Artesunate versus Quinine in the treatment of severe falciparum malaria in African children (AQUAMAT) trial had an overall mortality of 6–8.5% ^4^. The trial population were selected pragmatically (defined by parasite-positivity and the admitting clinician’s desire to use parenteral rather than oral antimalarials) for generalisability of the results. However, mortality in children with severe malaria would almost certainly be substantially higher outside a Good Clinical Practice (GCP)-run trial and outside selected centres with a strong track record of research; and with more stringent clinical criteria defining severe malaria ^5^. Thus, there is substantial potential for clinical trials addressing the safety and efficacy of adjuvant supportive therapies to not only close existing gaps in the severe malaria treatment algorithm, but also substantially improve outcomes ^6^.

Children with severe malaria and bacterial co-infection, largely due to enteric Gram-negative organisms with a predominance of non-typhoidal salmonella (NTS) species, have substantially higher rates of in-hospital and post-discharge mortality ^7^. An estimated one third of all severe malaria deaths in African children are attributable to bacterial co-infection ^8^. Current guidance and evidence for treating co-infection in children is lacking, and there is no consensus on the dosage or length of treatment required. Identifying which children genuinely have bacterial co-infection is practically impossible. The indiscriminate use of antibiotics is both financially costly and may perpetuate the rise of antibiotic resistance. Establishing which children with malaria are at greatest risk of bacteraemia is critical to pragmatically inform a policy for targeted antibiotic therapy that could substantially reduce malaria-associated mortality while minimising the risks of excess antibiotic prescribing.

### Bacterial co-Infection in severe malaria

We conducted a systematic review in 2013 examining studies among children with malaria admitted to hospitals or outpatient clinics in sub-Saharan Africa reporting invasive bacterial infection (IBI) ^9^. We identified a total of 25 studies across 11 African countries which fulfilled the inclusion criteria. These comprised of 20 cohort analyses, two randomised controlled trials and three prospective epidemiological studies. We initially compared the prevalence of IBI in 20,889 children who were hospitalised with all-severity malaria to 27,641 children hospitalised with a non-malarial febrile illness. The mean prevalence of IBI was 5.58 (95% CI 5.5 to 5.66%) in children with malaria and 7.77% (95% CI 7.72 to 7.83%) in non-malaria illness. In the meta-analysis involving 10 studies (n=7,208) of children with severe malaria the mean prevalence of IBI was 6.4% (95% confidence interval (CI) 5.81 to 6.98%). Ten of the 25 studies reported mortality stratified by bacterial infection status. Case fatality was higher at 81 of 336, 24.1% (95% CI 18.9 to 29.4) in children with malaria/IBI co-infection compared to 585 of 5,760, 10.2% (95% CI 9.3 to 10.98) with malaria alone. In seven of 14 hospital studies, NTS was the commonest isolate and one other study listed enteric Gram-negative organisms as the most common isolates in malaria-infected children ^9^. Thus, enteric Gram-negative organisms, and NTS in particular, cause the vast majority of bacteraemia co-infections ^9^. Moreover, the poor sensitivity of blood cultures (due to low blood volumes in children and previous antibiotic use) means that the true prevalence of bacteraemia co-infection is likely to be significantly higher and ‘nosocomial bacteraemia’ will be acquired in a further 2–3.5% of children receiving a transfusion (commonly prescribed in severe malaria) due to poor quality.

### Current management recommendations

Current recommendations indicate that antibiotics should be given to ‘all children with suspected severe malaria in areas of moderate and high transmission until a bacterial infection is excluded… and should be based on culture and sensitivity results or, if not available, local antibiotic sensitivity patterns’ ^10^. However, many hospitals in Africa lack culture facilities or access to such data; thus, best practice is not obvious. In one study almost 50% of bacterial isolates were resistant to the antibiotics most commonly recommended (chloramphenicol and gentamicin for empirical use) ^11^. In the specific case of NTS, the efficacy of gentamicin is doubtful and susceptibility testing unreliable due to this infection’s intracellular nature. For newer and broad-spectrum antimicrobials, apart from financial considerations, there are concerns that over-use could lead to resistance. A systematic review of studies and trials of supportive therapy in severe malaria was undertaken. Whilst definitive evidence for parenteral antimalarial treatment has now been provided ^4, 10^, progress on supportive therapeutics has been limited. To date, of the 34 clinical trials involving adjunctive therapies in severe malaria, including 20 (59%) in African children, none of these trials have targeted bacterial co-infection ^6^. Searching ClinicalTrials.gov and ISRCTN we found no trials addressing anti-microbial treatment of bacterial co-infection in severe malaria.

### Justification for azithromycin for malarial sepsis

There are several reasons why we consider that azithromycin represents an attractive option in such an approach. Given the worldwide threat of antimicrobial resistance, the underlying principle is to test the narrowest spectrum antibiotic that would be practical, generalisable and plausibly have reasonable efficacy. There are several reasons for selecting azithromycin as the targeted antimicrobial invention. Primarily, it is active against NTS, the commonest cause of bacterial co-infection in severe malaria ^9^, against which currently recommended antimicrobials are ineffective (e.g. chloramphenicol, ampicillin/gentamicin combination) ^11^. It is also active against a range of other gram-negative and gram-positive organisms, although it is less active against *Escherichia coli* and Klebsiella spp. The alternative (effective) antimicrobial therapies we considered, given that they would likely be active against NTS ^12^, were, first, a full course (~seven days) of parenteral third generation cephalosporins. This has feasibility concerns as it would prolong hospital stay (median four-five days), and would be difficult (and potentially unethical) to blind. In contrast to macrolides, which are not widely used in other childhood diseases ^13^, adding a major new indication for third-generation cephalosporins (severe malaria) also risks widening their use even further, and thus increasing the threat of antimicrobial resistance to a second-line antibiotic recommended for other common childhood diseases. Quinolones (e.g. ciprofloxacin) are another alternative strategy, given their superior antimicrobial activity against Salmonella species ^13^, *E. coli* and Klebsiella spp. However, resistance is generated through single mutations in the housekeeping genes gyrA or gyrB; and can be detected shortly after administration in healthy volunteer studies, suggesting resistant variants are commonly present at low levels or easily acquired ^14^. A recent study found pathogenic ciprofloxacin-resistant *E. coli* were highly prevalent in healthy children and their mothers ^15^, raising the possibility that widespread use in children with severe malaria without evidence of bacterial infection could have serious unintended consequences. Moreover, in most countries the use of fluoroquinolones in children is relatively contraindicated (due to concerns over musculoskeletal side-effects) except for use in multi-drug resistant infections where there are no suitable alternatives ^13^. Finally, although cephalosporin and quinolone resistance rates are currently relatively low in relevant organisms in some African countries ^12^, they are much higher in Asia ^13^, limiting generalisability of the trial’s findings worldwide.

Other favourable properties of azithromycin include:

1.   It is licenced for use and has a good safety profile in children.

2.   Azithromycin’s pharmacokinetic (PK) properties include extensive tissue distribution, prolonged phagocyte concentrations, and a longer elimination half-life (~50hours) than other macrolides, thus enabling once-daily dosing (versus more frequent dosing required for clindamycin) ^16^.

3.   Its long half-life is also likely to protect children at increased risk of NTS bacteraemia following a malaria episode ^17^.

4.   An accumulating body of evidence indicates some macrolides have beneficial properties independent of their antimicrobial effects ^18^. It is thus plausible that azithromycin may also have a beneficial effect on immune activation and gut inflammation.

5.   A further consideration is that azithromycin is a weak antimalarial ^19^, and has proved effective in treating *P. falciparum* malaria in early phase trials when used in combination with faster-acting antimalarials.

6.   Dispersible azithromycin is currently widely available at an affordable price, costing 0.40 euros per 100mg dose in an oral formulation. i.e. two-six Euros per five-day paediatric treatment course.

### Target population

Current recommendations indicate that antibiotics should be given to ‘all children with suspected severe malaria in areas of moderate and high transmission until a bacterial infection is excluded’ ^10^. However, WHO definitions of severe malaria are very broad, incorporating high parasitaemia as a single criterion, and are thus applicable to a large proportion of paediatric admissions in such regions, with a relatively overall low case fatality (1–2%) ^20^, who are unlikely to benefit from antimicrobial therapy as a group. For this study (and in a future trial) we aim to target antibiotics to children with the highest risk of bacterial co-infection, meeting ‘Teule’ criteria ^11^: that is, with malaria (positive blood film or ParacheckTM rapid diagnostic test, RDT); temperature > 38°C or < 36°C; and ≥1 of prostration, respiratory distress, haemoglobin (Hb) <5g/dl or HIV. These criteria identified 85% of bacterial co-infections, with a three-fold higher mortality than children admitted with malaria without these criteria.

### Credibility: can antibiotics be targeted?

A major legitimate concern is whether future management guidelines can be implemented which try to reduce the overuse of antibiotics where microbiology services are poor or non-existent, thus an approach to targeting only those with IBI is not feasible in much of Africa. Within the context of this study and to inform a future trial, we aim to investigate approaches (that may be generalizable in future) to targeting antibiotics. We propose to use (i) simple clinical criteria: proposed by Nadjm *et al.* ^11^, the Teule criteria identifies 85% of malaria cases with culture-proven bacteraemia: a pathogen was isolated from 20% children meeting these criteria. Simple and objective criteria identifying those at risk would be more generalizable outside research centres and would be validated in this study. (ii) Potential biomarkers: even when quality-controlled blood-culture facilities are available, low sensitivity (due to frequent pre-hospital antibiotic therapy, low culture volumes from children and low bacterial density) and long times to culture-positivity (typically two-three days) mean that alternative approaches for identifying concurrent gram-negative bacteraemia would be highly valuable.

## Protocol

Trial Registration ISRCTN49726849 (registered on 27 ^th^ October 2017).

Protocol Version 1.2: Date 9 ^th^ October 2018.

### Justification for the study

This trial, designed to inform a key research gap, proposes targeted and appropriate antimicrobial treatment of children with severe malaria at greatest risk of bacterial co-infection. Incorporated in the design are experimental data to demonstrate that the mechanisms that azithromycin is targeting are biologically reasonable, that the dosing is providing the right exposure, and that there is a pharmacokinetic-pharmodynamic (PKPD) link between azithromycin exposure and the potential mechanisms for reducing both short-term and microbiological cure (seven-day) and longer-term mortality (28-day). Comparison with a control group will also enable us to identify whether antimicrobial treatment can be further targeted.

At present pharmacological data are lacking on the correct dosage of oral azithromycin for treatment of bacterial co-infection in severe malaria in African children. We will therefore conduct a Phase I/II trial comparing three doses of azithromycin: 10, 15 and 20 mg/kg (prescribed for feasibility by weight-bands) spanning the lowest to highest mg/kg doses demonstrated to be equally effective as parenteral treatment for other salmonellae infection ^21^.

### Our hypotheses

1. Azithromycin given to children once-daily for five days in addition to standard treatment of severe malaria (including anti-malarials) can provide adequate dosing in children admitted to hospital with severe malaria.

2. Children with severe malaria and culture-proven bacteraemia can be accurately identified using clinical criteria alone or in combination with a rapid diagnostic biomarker tests, in comparison with a control cohort of children hospitalized with severe malaria but not meeting Teule criteria, at low risk of bacterial co-infection.

## Objectives

### General objectives

Our principal objectives are to:

(i)   establish the appropriate dose of oral dispersible azithromycin as an antimicrobial treatment for children with severe malaria;

(ii)   investigate whether antibiotics can be targeted to those at greatest risk of bacterial co-infection using clinical criteria alone or in combination with a rapid diagnostic biomarker test.

### Specific objectives

(i)   To conduct a Phase I/II trial comparing three doses of azithromycin: 10, 15 and 20 mg/kg (prescribed for feasibility by weight-bands) spanning the lowest to highest mg/kg doses demonstrated to be equally effective as parenteral treatment for other salmonellae infection.

(ii)   To determine, via PKPD modelling, the optimal azithromycin dose in severe malaria, and investigate associations between azithromycin exposure and pathogen susceptibility (minimum inhibitory concentration, MIC) with treatment outcome. Outcome measures will consist of changes in C-reactive protein (CRP), a putative marker of sepsis at 72 hours (continuous) and microbiological cure (seven-day) (binary), alone and as a composite with seven-day survival, while providing preliminary data on longer-term survival (to day 90).

(iii)   To evaluate whether a combination of clinical, point-of-care diagnostic tests, and/or biomarkers can accurately identify the sub-group of severe malaria with culture-proven bacteraemia by comparison with a control cohort of children hospitalized with severe malaria but not meeting Teule criteria, at low risk of bacterial co-infection.

## Methods

### Study site

The trial will be conducted in Mbale Regional Referral Hospital (MRRH), Eastern Uganda, where we already have an on-going collaborations ^22, 23^, in an area with hyperendemic malaria and paediatric admissions of >20,000/year, approximately 50% with malaria parasitaemia. MRRH is now an established research hub of the KEMRI-Oxford-Wellcome Trust Major Overseas Programme, assisted by trial, data and laboratory management from Kilifi, Kenya.

### Study design

A Phase I/II open-label trial comparing three doses of azithromycin: 10, 15 and 20 mg/kg (prescribed for feasibility by weight-bands) spanning the lowest to highest mg/kg doses demonstrated to be equally effective as parenteral treatment for other salmonellae infection.

## Study populations

Children will be considered eligible for enrolment in this trial if they fulfil all the inclusion criteria and none of the exclusion criteria. 

### Inclusion criteria

*Cases:*

105 children aged six months to 12 years at admission to hospital with *P. falciparum* malaria (on either blood film or ParacheckTM RDT) and all of the following:

i) Axillary temperature >38°C or <36°C.

ii) Teule severity criteria ^11^: any one of the following: prostration; respiratory distress; severe anaemia (haemoglobin<5g/dL) or HIV infection.

iii) Parents willing/able to provide consent.

### Controls

The control cohort of children (n=50) are those hospitalised with severe malaria but not meeting Teule criteria (i.e., low risk of bacterial co-infection) whose parents are willing/able to provide consent.

### Exclusion criteria (cases only)

Major contraindications to azithromycin, e.g. strong existing clinical diagnosis of QT-prolongation. Concomitant use of interacting drugs: drugs that may cause QT-prolongation or drugs that may cause a pharmacokinetic interaction with azithromycin, like strong CYP3A/P-GP inducers and concomitantly administered antacids. 

## Sampling

### Sample size determination

A formal sample size was not calculated. Phase I/II trials are an important step in the assessment of new or existing interventions providing the first data on the feasibility of using a product in a new application. They assess the effect of products (how well they work), and the side effects they may have. They are not usually designed to compare superiority of different products or interventions.

The aim is to generate pilot efficacy data on the optimal azithromycin dose for children with severe malaria, which will inform the design of a later larger Phase III trial. The numbers required to address the trial objectives are therefore balanced against the exposure of children in these settings to a therapeutic intervention (dose) for which there are limited data to date.

The overall sample size for the trial will be 105 children randomised 1:1:1 to receive 10, 15 or 20mg/kg azithromycin (based on weight-bands). This is sufficient for the PKPD sampling and modelling to determine an optimal dose in children with severe malaria using change in CRP at 72 hours, and microbiological cure (seven-day) alone and with seven-day survival. This is under the assumption that 20% of enrolled children (meeting Teule severity criteria) will have bacteraemia and that 80% of these infections will be caused by NTS or other enteric gram-negative organisms based on previous research. 

Based on Monte Carlo simulations, we expect that all dose groups should reach the population reference target (adult) AUC24h of at least 3.4 h*mg/L ^24^, although there are no data on absorption in severe malaria, which is characterised by impaired gut function ^25^, hence the need for this pilot. Along with the 105 children with Teule criteria we will enrol 50 children meeting other inclusion criteria but without Teule criteria (and without any exclusion criteria) into the control group.

### Study methods and procedures

Eligible children will be identified by the nurse and clinician on duty and registered in the eligibility screening log. A member of the trial team will then perform a rapid structured assessment of heart rate, oxygen saturation (pulse oximetry), respiratory rate, axillary temperature, blood pressure, markers of shock (capillary refill time, pulse volume and assessment of lower limb temperature) and severity (conscious level and respiratory distress). Children who are potentially eligible with suspected severe malaria will have a rapid bedside malaria test (detecting *P. falciparum* histidine-rich protein 2, HRP2) to determine malaria status and eligibility.

### Randomisation procedure

Randomisation lists will be generated and kept at the Medical Research Council (MRC) Clinical Trials Unit at University College London, London. The randomisation envelopes will be prepared before the trial, using the lists at the Clinical Trials Facilty, KEMRI Wellcome Trust Research Programme (KWTRP), Kilifi, Kenya. These will be opaque and sealed and will contain a card with allocation. Children will be randomised (1:1:1) to receive 10, 15 or 20 mg/kg azithromycin (based on weight-bands). The cards will be numbered consecutively and opened in numerical order. Clinicians will be aware of the treatment-group dose assignments, but the laboratory tests are to be performed in a blinded manner.

### Consent process

Once eligibility has been confirmed, authorised trial staff will approach parents/guardians to invite their child to take part in the trial. An information sheet will be provided to the parent/guardian in their usual language containing details of the TABS trial. The sheet will be read aloud to those who are unable to read. The doctor/nurse will check that the information has been fully understood and parents/guardians will be encouraged to ask questions they may have about their child’s participation in TABS trial. The information sheet and consent includes details of the clinical trial, follow up and additional biological samples taken for the trial and permissions for sample storage. Where possible, prospective written informed consent will be sought from parents/guardians who will then be asked to sign the consent form. Consent will include permission for the collection of admission and follow-up blood samples for later aetiological investigations. If parents/guardians are unable to sign, a thumbprint will be taken in lieu of a signature. A copy of the consent form will be given to the parent/guardian, the original placed in the patient’s medical notes, and a copy kept in the investigator site file. A number of children will present as emergencies where delay in study enrolment, and thus treatment, will not be practical or indeed humane. We will use a modified form of deferred consent; used in the FEAST trial ^22^ and for which we have received ethical approval. It proposes to use a ‘two-stage’ consent process in this circumstance ^26^. Verbal assent will be sought from parents or guardians by the admitting medical team if it is considered that the full consent process would significantly delay treatment allocation, and consequently could be detrimental to the child’s health. Full consent will be sought once the child’s clinical condition has been stabilized. Caregivers will be provided with a brief verbal description of the trial and will be given the opportunity to “opt out”. As in the FEAST trial, if following an assent process a child died prior to full written consent, full consent would not be sought. This process of emergency consent was approved for FEAST and has been subsequently approved for use in a TRACT transfusion trial in Uganda and Blantyre ^23^.

### Treatment allocation

**Figure 1.**
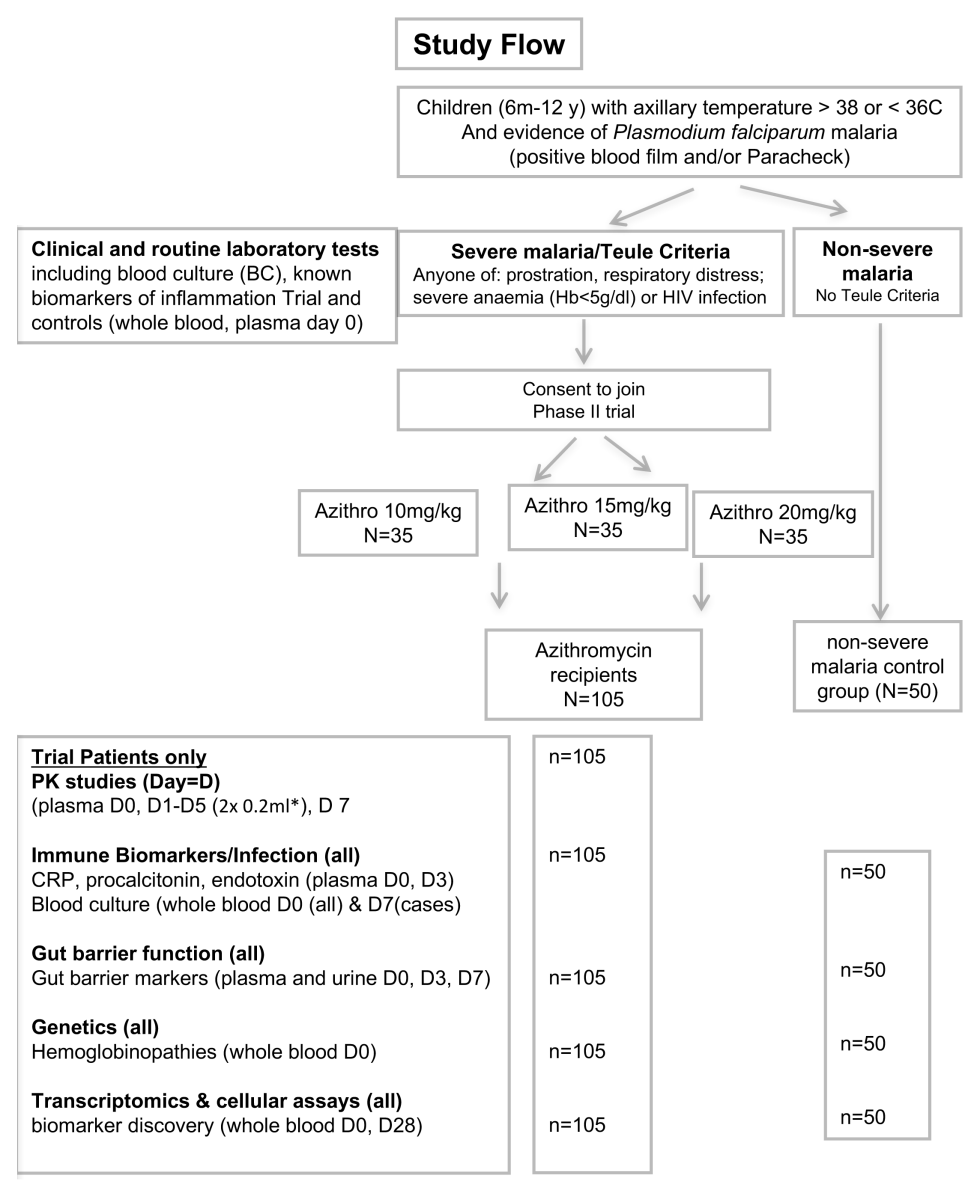
Trial flow. PK, pharmacokinetics; CRP, C-reactive protein. * Days 1-5 inclusive.

Following consent, children will be randomly allocated to the treatment arms. Children, on admission to hospital with severe malaria ( *see Inclusion criteria above*) eligible for the trial will be randomised to one of three doses of adjunctive once daily oral azithromycin doses, which will be given for five days in addition to a three-day course of parenteral artesunate. The comparator cohort with non-severe malaria will receive usual standard of care (oral artemisinin combination therapy). See Study Flow ( Figure 1).

### Pragmatic dosing chart

Rather than calculate azithromycin doses based on body weight (which in practice is poorly implemented), the trial will use weight bands (see Table 1) all using 100mg dispersible tablets, which are more practical and generalizable outside of clinical trials.

**Table 1.** Pragmatic dosing chart for dispersible azithromycin.

	10 mg/kg	100mg tabs	15 mg/kg	100mg tabs	20 mg/kg	100mg tabs
Weight (kg)	Weight x10	Tab size	Weight x15	Tab size	Weight x20	Tab size
5.5 - 6.4	55-64	0.5	82.5-97	1	110-129	1
6.5 - 7.4	65-74	0.5	97.5-112	1	130-149	1.5
7.5 - 8.4	75-84	0.5	112.5-127	1	150-169	1.5
8.5 - 9.4	85-94	1	127.5-142	1.5	170-189	1.5
9.5 - 10.4	95-104	1	142.5-157	1.5	190-209	2
10.5 - 11.4	105-114	1	157.5-172	1.5	210-229	2
11.5 - 12.4	115-124	1	172.5-187	1.5	230-249	2.5
12.5 - 13.4	125-134	1	187.5-202	2	250-269	2.5
13.5 - 14.4	135-144	1.5	202.5-217	2	270-289	2.5
14.5 - 15.4	145-154	1.5	217.5-232	2	290-309	3
15.5 -16.4	155-164	1.5	232.5-247	2.5	310-329	3
16.5 - 17.4	165-174	1.5	247.5-262	2.5	330-349	3.5
17.5 - 18.4	175-184	1.5	262.5-277	2.5	350-369	3.5
18.5 - 19.4	185-194	2	277.5-292	3	370-389	3.5
19.5 - 20.4	195-204	2	292.5-307	3	390-409	4
20.5 - 21.4	205-214	2	307.5-322	3	410-429	4
21.5 - 22.4	215-224	2	322.5-337	3	430-449	4.5
22.5 - 23.4	225-234	2	337.5-352	3.5	450-469	4.5
23.5 - 24.4	235-244	2.5	352.5-367	3.5	470-489	4.5
24.5 - 25.4	245-254	2.5	367.5-382	3.5	490-509	5
25.5 - 26.4	255-264	2.5	382.5-397	4	510-529	5
26.5 - 27.4	265-274	2.5	397.5-412	4	530-549	5.5
27.5 - 28.4	275-284	2.5	412.5-427	4	550-569	5.5
28.5 - 29.4	285-294	3	427.5-442	4.5	570-589	5.5
29.5 - 30.4	295-304	3	442.5-457	4.5	590-609	6
30.5 - 31.4	305-314	3	457.5-472	4.5	610-629	6

This weight-band dosing has been developed based on existing data ^27^ and will be validated by the nested PKPD sub-study (as has been done in several HIV trials). Children generally tolerate a wide range of azithromycin doses (5-20mg/kg); however, when given in combination with artesunate, doses of 20mg/kg more commonly resulted in vomiting (versus artemether-lumefantrine) ^28^. Based on Monte Carlo simulations, we expect that all dose groups should reach the population reference target (adult) AUC24h of at least 3.4 h*mg/L ^24^, although there are no data on absorption in severe malaria, which is characterised by impaired gut function ^25^, hence the need for this pilot.

### Trial assessment schedule

Children will be intensively monitored during admission by the clinical team at least eight hourly in the first 72 hours following recruitment, and daily thereafter. Locator maps and contact numbers will be obtained to facilitate follow-up. All participants will then be seen at seven-days, 28-days and 90-days post-admission at outpatient clinics attached to each centre for evaluation of morbidity and toxicity. Any patient not returning for a study visit will be traced for vital status ascertainment ( Table 2).

**Table 2.** Schedule of clinical assessments and laboratory investigations.

Procedure	Rand *	0m	30m	60m	90m	2hr	4hr	8hr	24hr	48hr	Daily	Disc *
**Source documents**	X	X	X	X	X	X	X	X	X	X	X	X
Clinical notes (doctor) ^$^	X	X	X	X	X	X	X	X	X	X	X	X
Bedside observations (nurse)	X	X	X	X	X	X	X	X	X	X	X	
Haemoglobin (Hb)	X							X	X	X		
Lactate	X							X	X			
Glucose	X					X		X	X			
Prescription chart	X								X	X	X	X
Anthropometry	X											X
Case report form	X	X	X	X	X	X	X	X	X	X		X
Diagnosis ^¢^	X											X
Drugs received									X	X		X
Transfusion and iv fluids charts			X	X		X	X	X	X	X		X
Locator details	X											X
Investigations ^&^	X					X		X	X	X	X	
Serious adverse events		X	X	X	X	X	X	X	X	X	X	X
Discharge medication												X

* Rand: At randomisation; Disc: On Discharge from hospital ^$^ Additional reviews by doctors and nurses will be conducted and recorded, where clinically indicated. ^&^ Laboratory tests and bedside tests including glucose and lactate at time points shown; plus any additional investigations as clinically required eg chest-Xray ^¢ ^Working diagnosis at admission and final diagnosis at discharge.

### Blood sampling schedule

Following consent and randomisation, blood samples will be taken for the following investigations: full blood count, urea and electrolytes, lactate, glucose, malaria status (malaria slide), blood culture and other clinically indicated investigations (not required by the study protocol). Urine will be taken as soon as possible after trial enrolment for multi-stick analysis for assessment of cola or red colour urine and additional storage for later analysis of gut barrier function. In accordance with national guidelines, HIV testing will be performed during admission procedures since this is one of the eligibility criteria. Pre- and post-test counselling will be done in accordance with routine practice. At admission, a venous blood sample will be collected into Heparin and EDTA tubes and stored for subsequent cytokine and biomarker assays, parasitological ( *P. falciparum* HRP2), genetic studies (including sickle cell and glucose-6-phosphatase deficiency status) and immune activation studies (transcriptome and cellular assays). For the latter, red and white cell pellets will be prepared and stored. Further blood samples will be taken at 72 hours, seven days and 28 days (for biomarker assays and immune activation studies); and at day seven for repeat blood culture (see Table 3). Changes in inflammatory markers (including CRP and procalitonin, PCT) will be measured retrospectively at 72 hours and day seven and microbiology cure measured at day seven (from blood cultures and using non-culture based molecular diagnostics on stored blood).

**Table 3.** Detailed laboratory investigation schedule.

Day from recruitment	D0	D1	D3	D7	D28
Blood culture	3 ml			3 ml (cases)	
PK studies (cases only)	2 x 0.25 ml			1 x 0.25 ml	
Whole blood (Endotoxin)	1.5 ml		1.5 ml	1.5 ml	
Whole blood EDTA (genetics)	1 ml				
Whole blood (transcriptomics, CRP, procalcitonin, calprotectin, gut barrier function)	4.5 ml		2 ml	2 ml	4.5 ml
**Total blood volume research**	**10.4 ml**	**0.5 ml**	**3.5 ml**	**7 ml**	**5 ml**
Urine	x	x	x		

PK, pharmacokinetics; CRP, C-reactive protein.

### Clinical management and monitoring

All trial patients will receive standard of care including (intravenous or oral) anti-malarial drugs following national guidelines, based on WHO syndromic patient management ^29^. We will collect data on all administered drugs. Antipyretics, anticonvulsants and treatment for hypoglycaemia will be administered according to nationally agreed protocols. If required, maintenance fluids will be run at 3-4 ml/kg per hour irrespective of age until the child can drink and retain oral fluids. As the trial is designed to target the use of ‘appropriate’ antimicrobial treatments, clinicians will be free to prescribe antibiotics other than cephalosporins, quinolones or macrolides on admission day two based on the child’s clinical condition and according to country guidelines. On day three (or earlier if results available) clinicians will be permitted to prescribe broad-spectrum antimicrobials guided by the admission blood culture results (species and/or susceptibility). The Uganda severe malaria guidelines give no specific guidance on the use of antibiotics in severe malaria. Children or their carers will be provided with an appropriate supply of azithromycin drugs if discharged before day five.

The clinical coordinator is responsible for ensuring the discharge check-list is complete and for chasing up inpatient notes at discharge. Any relevant information, especially with regard to date of discharge, serious adverse events (SAEs), treatments, blood transfusions, use of intravenous fluids, oxygen or non-routine treatments or investigations, will be recorded. Children will be assessed on day seven, day 28 and day 90 following recruitment. The parent/guardian will receive a follow up invitation on a card. A symptom checklist and targeted physical examination will be performed at each clinic visit post-discharge. Medical history since last visit including hospital re-admissions, transfusions and grade three or four adverse events related to the antibiotic interventions including severity and likely relationship of any adverse events will be documented by a doctor. Any child lost to follow-up before 90 days will be traced for vital status. In order to minimise losses to follow-up, locator data (maps and identifiable landmark) and mobile phone numbers will be taken on discharge and verified at every review. During the 90 day study period attempts will be made to contact the patient via phone (if available) and to follow-up with home visits, if clinic visits are not attended. In the statistical analysis, a patient will be regarded as ‘lost to follow-up’ if they were not seen in clinic at the day 90 visit and were not known to have died.

### Trial products, storage and accountability

The product to be tested in the trial is azithromycin 100 mg dispersible tablets, which are a commercial product and were obtained for the trial as a donation from Cipla Pharmaceuticals, Mumbai, India. Azithromycin is approved for use in children in Uganda where the trial will take place. Cipla Ltd is fully GMP compliant and has provided full certificates for the manufacture of these products. Azithromycin will be stored at room temperature and not exceeding 30°C (86°F). It will be stored in these conditions for three years as packaged. The trial coordinator at the site will maintain accountability logs for the antibiotic intervention (standard and interventional). These will be kept securely until verified by the external monitor’s visit.

## Sub-studies

### PKPD study

For PK analysis, model-based sampling strategies will enable the description of both intra- and inter-patient variability. A maximum of 3x 0.2ml samples will be taken from each subject on two occasions between day one to five around meaningful time points to describe the PK throughout a dosing interval plus one sample at follow up on day seven to determine the elimination of azithromycin after end of treatment (a total of seven samples per patient). In the first year we will develop highly sensitive bioanalytical assays using ultra performance liquid chromatography coupled with tandem mass spectrometry (UPLC-MS) for determination of azithromycin in stored microvolumes of plasma (analysis performed at Radboud University, Nijmegen). 

Initially a population PK model will be developed for azithromycin and will investigate possible covariates for its PK. This will be followed by an investigation of the relationship between plasma PK and PD using the developed PK model, known MIC-values since the area under the curve (AUC):MIC ratio is the pharmacodynamic driver for azithromycin ^16, 30^ and the treatment outcomes (changes in CRP at day three and microbiologic cure and/or mortality at day seven). Based on Monte Carlo simulations, we expect that all dose groups should reach the population reference target (adult) AUC24h of at least 3.4 h*mg/L ^24^, although there are no data on absorption in severe malaria, which is characterised by impaired gut function ^25^, hence the need for this pilot. The different doses in 105 children provides variability in exposure so that we identify whether all doses are in the flat part of the exposure-response curve, in which case the lowest dose will be chosen. Other secondary endpoints include mortality at 48 hours, length of hospital stay (days), re-hospitalisation and other adverse events. Quality-control of stored bacterial isolates, non-culture (molecular) assessment of bacterial infection including the determination of MIC to inform PKPD studies will be batch-processed at KWTRP, Kenya.

### Biomarker and gut barrier function

In 105 trial participants and 50 children with controls (severe malaria without Teule criteria) we plan to investigate from admission samples the utility of a range of potential biomarkers including CRP, PCT ^31, 32^ and endotoxin ^25^, to identify whether any of the above markers, alone or in combination, can predict which at-risk children with severe malaria have bacterial co-infections to improve the sensitivity of clinical/laboratory criteria and enable antimicrobial therapy to be further targeted. We will investigate associations between pathological concentrations of these markers and clinical, parasitological and microbiological data in cases (meeting Teule criteria) and controls (not meeting Teule criteria) to test their accuracy for stratification in the main trial. The samples will also allow us to analyse markers of gut barrier dysfunction – a requisite for bacteraemia (intestinal fatty acid binding protein (I-FABP) and the ileal-bile acid binding protein (I-BABP) and establish whether these can accurately predict children with blood-culture proven bacteraemia alone or in combination with clinical criteria/RDTs. In addition, we will analyse the peripheral blood mononuclear cell transcriptome in a subset of children with and without bacterial co-infection to verify the significant changes in transcriptome we observed in a small pilot study of children with malaria and bacterial co-infection compared to children with malaria alone and to confirm observed changes on the cellular level in a second set of children ^33^. We will investigate whether changes in gene expression profile can be translated into rapid and affordable biological tests predicting children with bacterial co-infection with high specificity and sensitivity. This later analysis is subject to additional funding.

## Trial outcome measures

### Primary outcomes

•  Change in CRP, a putative marker of sepsis at 72 hours (continuous) and microbiological cure (seven-day) (binary), alone and as a composite with seven-day survival.

•  The changes in CRP between day 0 and day 3 as the main endpoint for the PKPD studies.

### Secondary outcomes

The secondary outcome measures are:

•  Mortality at 48-hours and longer-term survival (to day 28 and to day 90).

•  Length of hospital stay (days)

•  Re-admission to hospital by 90 days

•  Adverse events

•  The population PK of azithromycin and their relation (combined with pathogen susceptibility) with treatment outcome (PD).

### SAEs and interim analyses

SAEs will be reviewed immediately by a designated physician (SAE reviewer) and reported to the appropriate ethics and regulatory committees within one week. The Chief Investigator will inform the Trial Steering Committee (TSC) and Data Monitoring Committee (DMC) for review on a regular basis (as deemed necessary).

### DMC

An independent DMC (see composition at the end of the protocol) will review data on enrolment, safety, adherence to the trial protocol and efficacy at regular intervals and in strict confidence. The terms are covered in the DMC charter, signed by the chair and trial statistician. There are no fixed ‘stopping rules’. The DMC will receive and review information on the progress and accruing data of the trial and provide advice on the conduct of the trial to the TSC. The DMC would inform the Chair of the TSC if, in their view the results are likely to convince a broad range of clinicians, including those supporting the trial and the general clinical community, that, on balance, one trial arm is clearly indicated or contraindicated for all participants or a particular category of participants. The DMC is comprised of a chair and two other independent members. None declared conflicts of interest.

### Trial monitoring

The Mbale site will use a web enabled trial database and will be responsible for data entry and local trial management. The site will retain the original case report forms (CRFs). Data stored on the database will be checked for missing or unusual values (range checks) and checked for consistency within participants over time. If any such problems are identified, the site will be contacted and asked to verify or correct the entry. Changes will be made on the original CRF and entered into the database at the site. Kilifi Clinical Trial Facility will also send reminders for any overdue and/or missing data with the regular inconsistency reports of errors.

This trial will be monitored according to a monitoring plan which will set out the frequency of visits, the degree of source document verification against the CRFs and the requirements for triggered on-site monitoring visits. This plan will also detail the procedures for review and sign-off. The monitoring will adhere to the principles of ICH GCP.

The site initiation visits will include training in the trial procedures, as well as practical training in administration of trial interventions, reporting guidelines for adverse events of study interventions as well as other trial procedures. All staff at sites involved in the trial will receive formal training in GCP through a dedicated training programme during site initiation visit and will also be required to complete an on-line course. 

The Clinical Trial Facility in Kilifi oversees standards and quality of all trials conducted through the KWTP and through its monitoring systems and standard operating procedures are organised to ensure that all sites can be monitored with equal independence and rigor. All monitors will be appropriately qualified and trained.

At each monitoring visit the monitors will:

▪   verify completeness of Trial Master File

▪   confirm adherence to protocol

▪   review eligibility verification and consent procedures

▪   look for missed clinical event reporting

▪   verify completeness, consistency and accuracy of data being entered on CRFs

▪   evaluate drug accountability

▪   provide additional training as needed

The monitors will require access to all patient medical records including, but not limited to, laboratory test results and prescriptions. The investigator (or delegated deputy) should work with the monitor to ensure that any problems detected are resolved.

### Data management

All clinical and laboratory data will be recorded in the CRF and stored with a unique serial number identifier. Data will be entered (double data entry) onto OpenClinica. All data will be regularly backed up and backup copies stored both on and off site. Paper records will be archived in locked cabinets. These cabinets will have limited access with prior authorisation (by the site principal investigator). All data will be partially anonymised prior to presentation or publication of any results. Study participants will be identified by a unique subject identification number but patient identifiable information will not be recorded on the study database in compliance with GCP requirements. The data will be examined for inconsistencies during the trial by the statistician and fed back to study sites for corrections following GCP procedures.

### Confidentiality

All clinical data will be held confidentially, and personal identifiers will be removed before analysis of the data and presentation of the results.

### Data sharing

After completion of the study, requests for data access from researchers outside the study team will be considered by the trial management team and clinical trials unit (Data Governance Committee), and where indicated, requestors will be asked to develop scientific protocols for approval of secondary analyses. The potential to share data will be included in the participant Information and Consent Form (see extended data section) ^34^.

### Statistical analysis

The primary analysis for the trial will describe baseline parameters (stratified by study arm) and compare primary and secondary end points by trial arm. The primary analysis will be by intention to treat and secondary analysis will include those treated per protocol (i.e., receiving five days of azithromycin).

The co-primary outcomes are change in CRP (which will be analysed using normal linear regression adjusting for baseline, using appropriate transformations) and microbiological cure (seven day) alone and as a composite with seven-day survival (which will be analysed using logistic regression and exact tests as a binary outcome). Analysis of adverse events and re-admission will use time-to-event methods through day 90 counting in-hospital death as a competing risk. Changes in CRP and other inflammatory markers will be analysed using normal linear regression (potentially on log-transformed data), using generalised estimating equations to jointly model changes at 72 hours and day-seven. Adverse events will also be summarised by body system.

### Ethics statement

Ethical approval has been obtained Mbale Regional Hospital Research Ethics committee (MRRH_REC 095/2017) and from Imperial College Research Ethics Committee (17IC3965), the sponsor of the study. The trial was registered on ISRCTN (ISRCTN49726849) on 27 ^th^ October 2017 and updated on 12 ^th^ August 2020 (detailing delay in start of trial and new start date).

### Safety

The randomised trial will be conducted in children who are most likely to benefit from the treatment. We will minimise the risks of cannula insertion and phlebotomy by pretrial training in phlebotomy technique and regular cannula site inspection which is included in a SOP. We have calculated that no more than 1ml/kg of blood will be drawn for research purposes at any one time.

### Benefits

Children with severe malaria often develop complications such as convulsions, severe anaemia and hypoglycaemia which through close clinical monitoring will be identified at the earliest opportunity and appropriate therapy initiated. Mbale Regional Referral Hospital trial has considerable experience with managing severe malaria and this will serve to minimise the risks to the patients in the trial. Pretrial training of the dedicated study team will include specific training on general management of severe malaria and its complications. A manual of operations will provide clear management guidelines as well as the details of trial conduct and procedures. Children enrolled in the trial will therefore receive a higher quality of care than those managed routinely.

## Plans for dissemination of the study outcomes

### Public engagement

Results from this trial will be disseminated locally through community meetings and national meetings with the wider healthcare professional community. These systems have been developed for dissemination of MRC FEAST and TRACT trial results and will be adapted to dissemination for the TABS PKPD trial.

### National and international policymakers

The site principal investigator has discussed the study with Ministry of Health staff. When the results are available, we will provide a summary briefing highlighting the trial results and what then next steps will be. Whilst the current study will go some way towards addressing whether azithromycin could be used as an adjunctive treatment in severe malaria a future trial should include a pragmatic design to ensure results are applicable to health services in Africa. The approach we propose may also have utility for syndromic management of other conditions, especially those whose clinical features overlap with severe malaria and in areas where microbiological diagnostic facilities are non-existent. 

## Discussion

Severe malaria remains a common cause of paediatric admission in many countries in Africa. Children who have bacterial co-infection are at high risk of poor outcome. Establishing which children with malaria are at greatest risk of bacteraemia is critical to pragmatically inform a policy for targeted antibiotic therapy that could substantially reduce malaria-associated mortality while minimising the risks of excess antibiotic prescribing. Short-course empiric therapy with early termination guided by culture results (to prevent development of resistance ^35^) is commonly practiced in high-income countries. However, poor microbiology services and overloaded health systems mean that this approach is not feasible in much of Africa and a trial designed around it would not be generalisable. Thus, in many parts of Africa antibiotic prescribing in children with severe malaria is highly variable across hospitals, with most physicians essentially using antibiotics “blind”, and thus are unable to follow WHO guidelines recommending stopping broad-spectrum antibiotics when bacterial infection has been ruled out. A more pragmatic approach would be to use a standard short-course of a moderately broad-spectrum antibiotic that is not already recommended for empiric use in a large number of other conditions with the potential for improving both early and post-discharge mortality. Relevant to a future trial and to current guidelines, is that many hospitals in Africa lack microbiological culture facilities, meaning best practice is not obvious given the clear threat posed by antimicrobial resistance ^36, 37^. To address this within the context of this study and to inform a future trial, we aim to investigate potential approaches (that may be generalizable in future) to targeting antibiotics where microbiological faciliaties are not present or reliable.

We favoured investigating azithromycin since one study found that almost 50% of bacterial isolates were resistant to the antibiotics most commonly recommended for empirical use ^11^. For NTS specifically, the efficacy of gentamicin is doubtful and susceptibility testing unreliable due to its intracellular nature ^17, 38^. Third generation cephalosporins (e.g. ceftriaxone) are the most widely used antimicrobials but resistance is becoming widespread. Moerover, few children will receive an ‘adequate’ therapeutic dose of cephalosporins, since NTS, the commonest cause of bacterial co-infection, requires ~seven days parenteral therapy thus requiring prolonged hospital stay (median four-five days) with economic implications. Further, adding a major new indication for third generation cephalosporins (severe malaria) risks substantially expanding their use, with downstream threats of AMR to a second-line antibiotic currently recommended for other common childhood diseases. Newer antimicrobials, apart from financial considerations, have similar concerns that over-use could lead to resistance. 

We therefore plan to study azithromycin because of its favourable microbiological spectrum, its inherent antimalarial and immunomodulatory properties and dosing and safety profile. Its long half-life is also likely to protect children at increased risk of NTS bacteraemia during the month following a malaria episode. At present pharmacological data are lacking on the correct dosage of oral azithromycin for treatment of bacterial co-infection in severe malaria in African children. The TABS trial will therefore conduct a Phase I/II trial comparing three doses of azithromycin: 10, 15 and 20 mg/kg (prescribed for feasibility by weight-bands) spanning the lowest to highest mg/kg doses demonstrated to be equally effective as parenteral treatment for other salmonellae infection. Treatment will be given over five days in children with severe malaria at highest risk of bacterial co-infection (meeting Teule criteria) in order to generate relevant PK data by sparse sampling during dosing intervals. The goal is to determine, via population PK modelling, the optimal azithromycin dose in severe malaria, and investigate associations between azithromycin exposure and potential mechanisms PKPD using change in CRP, a putative marker of sepsis at 72 hours (continuous) and microbiological cure (seven-day) (binary), alone and as a composite with seven-day survival, while providing preliminary data on longer-term survival (to day 90). We will also evaluate whether a combination of clinical, point-of-care diagnostic tests, and/or biomarkers can accurately identify the sub-group of severe malaria with culture-proven bacteraemia by comparison with a control cohort of children hospitalized with severe malaria but not meeting Teule criteria, at low risk of bacterial co-infection.

A future Phase III trial may therefore consider two strata: first comparing a pharmacologically-informed dosage of oral azithromycin to standard-of-care (largely third generation cephalosporins) in children at highest risk of bacterial co-infection and second comparing standard of care versus no antibiotics in children with severe malaria but minimal risk of bacterial co-infection to establish whether a policy for targeted antibiotic therapy could substantially reduce malaria-associated mortality while minimising the risks of excess antibiotic prescribing. Risk of co-infection could be determined by Teule criteria ^11^, but would also be informed by this pilot trial. The primary outcome would be mortality at either day seven, day 28 or day 90 mortality which is also be informed by this pilot trial (other timepoints would be secondary outcomes). Other secondary outcomes include mortality at 48 hours, length of hospital stay (days); subsequent hospital readmission; adverse events, faecal changes in antimicrobial resistance ^34^ and cost-effectiveness.

### Trial status

Trial enrolment started in January 2021. The delayed start of recruitment was due to the requirement for CIPLA to donate the trial drug which was mandated by the National Drug Authority (NDA), Uganda. Although we received support from CIPLA (2019) and the relevant approvals for importing the drug for the trial was then secured from NDA there was significant delays to importing the dispersible azithromycin for the trial and starting the trial due to the COVID epidemic. The trial completed recruitment on 4 ^th^ October 2021 and follow up on 4 ^th^. January 2022. The clinical trial data and PKPD are currently being analysed.

### Protocol version changes

Version 1.0 was the original protocol submitted for ethical approval to ICREC version 1.1 was given full approval on 11 ^th^ November 2017 following MRRH approval (27 ^th^ October 2017) of the revised version 1.0 Protocol (version 1.2: dated 9 ^th^ October 2018 was approved by ICREC and MRRH REC following minor wording changes as a result of regulatory review/approval.

## Roles and responsibilities

### Role of study sponsor and funders

The sponsor and funder played no role in in study design and will play no role in data collection, trial management, analysis and interpretation of data and manuscript preparation the decision to submit the report for publication.

### Trial Management Group

Kathryn Maitland, Peter Olupot-Olupot, Hellen Mnjalla, Diana Gibb Elizabeth C George and Roisin Connon

### Trial Steering Committee

Professor Elizabeth Molyneux, OBE (Chairman): College of Medicine, Blantyre Malawi; Dr Jane Crawley: University of Oxford; Dr Irene Lubega: Department of Paediatrics Makerere University College of Health Sciences, Uganda. 

### Data Monitoring Committee

Prof. Timothy Peto (Chair): University of Oxford; Dr Jennifer Thompson (Trial Statistician): London School of Hygiene and Tropical Medicine; and Professor Philippa Musoka: Makerere University College of Health Sciences, Uganda.

### Sponsor

Imperial College London is the main research Sponsor for this study. For further information regarding the sponsorship conditions, please contact the Head of Regulatory Compliance at:

Joint Research Office, Room 221b, Medical School Building, St Mary’s Campus, Norfolk Place, London, W2 1PG. Telephone: +44 (0) 020 7594 1872.

## Data Availability

No underlying data are associated with this article. Imperial College Research Data Repository: TABS extended data. https://doi.org/10.14469/hpc/8312 ^34^. Imperial College Research Data Repository: SPIRIT checklist for “Pharmacokinetics and pharmacodynamics of azithromycin in severe malaria bacterial co-infection in African children (TABS-PKPD): a protocol for a Phase II randomised controlled trial”. https://doi.org/10.14469/hpc/8311. Data are available under the terms of the Creative Commons Zero "No rights reserved" data waiver (CC0 1.0 Public domain dedication).
