# Supplementary material for: Pharmacokinetics and pharmacodynamics of azithromycin in severe malaria bacterial co-infection in African children (TABS-PKPD): a protocol for a Phase II randomised controlled trial
Source: Wellcome Open Res. 2023 Jan 10;6:161. Originally published 2021 Jun 23. [Version 2] doi: 10.12688/wellcomeopenres.16968.2 (PMC10382785; doi:10.12688/wellcomeopenres.16968.2)
Supplement: Peer review history

# Peer review: Reviewer response for version 2

**DOI:** 10.21956/wellcomeopenres.20769.r61999

**Published:** 2023-07-28

**Authors:** Nworgu Confidence ogechi

**Affiliations:** 1 Ignatius Ajuru University of Education, Port Harcourt, Rivers, Nigeria

This study aims to determine the appropriate dose of oral dispersible azithromycin for treating children with severe malaria and investigate whether antibiotics can be targeted to those at high risk of bacterial co-infection using clinical criteria and rapid diagnostic biomarker tests. The trial will compare three doses of azithromycin and collect pharmacokinetic data to determine the optimal dose. The study will also evaluate the use of clinical criteria, diagnostic tests, and biomarkers to identify severe malaria patients with bacterial co-infection. The results will inform the design of a Phase III trial. However, there are a few areas that could be adjusted;

1. The abstract does not clearly state the primary objective of the study, which is to determine the appropriate dose of oral azithromycin for treating African children with severe malaria and bacterial co-infection. It also does not clearly state the secondary objective, which is to investigate the use of clinical criteria and biomarker tests to identify children at the highest risk of bacterial co-infection.
2. The article does not provide detailed information on the study design, such as the number of participants, the inclusion and exclusion criteria, and the randomization process. This information is important for readers to understand the validity and generalizability of the study findings.
3. The article does not provide information on the statistical methods that will be used to analyze the data. This information is important for readers to understand how the study findings will be interpreted and to assess the validity of the conclusions.
4. The article briefly mentions that current evidence for treating co-infection is lacking and that there is no consensus on the dosage or length of treatment required. However, it does not discuss other potential limitations of the study, such as potential biases or confounding factors, which could affect the validity of the study findings.

However, while the study protocol is of interest and addresses an important clinical question, the article could be improved by providing more clarity and detail on the study design, statistical analysis, and potential limitations.

Is the study design appropriate for the research question?

Yes

Is the rationale for, and objectives of, the study clearly described?

Yes

Are sufficient details of the methods provided to allow replication by others?

Partly

Are the datasets clearly presented in a useable and accessible format?

Partly

Reviewer Expertise:

Malaria chemotherapy, Immunopathology

I confirm that I have read this submission and believe that I have an appropriate level of expertise to confirm that it is of an acceptable scientific standard.

# Peer review: Reviewer response for version 2

**DOI:** 10.21956/wellcomeopenres.20769.r54042

**Published:** 2023-01-23

**Authors:** Stephen J. Rogerson

**Affiliations:** 1 Department of Infectious Disease, Peter Doherty Institute for Infection and Immunity, University of Melbourne, Melbourne, Vic, Australia

I am satisfied with the authors’ responses to my review.

Is the study design appropriate for the research question?

Yes

Is the rationale for, and objectives of, the study clearly described?

Partly

Are sufficient details of the methods provided to allow replication by others?

Yes

Are the datasets clearly presented in a useable and accessible format?

Not applicable

Reviewer Expertise:

Malaria in pregnancy and childhood. Pathogenesis and immunity. Collaborations with clinical pharmacologists but o specific expertise.

I confirm that I have read this submission and believe that I have an appropriate level of expertise to confirm that it is of an acceptable scientific standard.

# Peer review: Reviewer response for version 1

**DOI:** 10.21956/wellcomeopenres.18730.r48482

**Published:** 2022-02-07

**Authors:** Stephen J. Rogerson

**Affiliations:** 1 Department of Infectious Disease, Peter Doherty Institute for Infection and Immunity, University of Melbourne, Melbourne, Vic, Australia

Dr Olupot-Olupot and colleagues present a protocol for a trial that recently commenced recruitment in Uganda, comparing different doses of oral azithromycin as adjunctive treatment for children with severe malaria. Although all doses would be expected to be effective if used parenterally for the commonest pathogen, non-typhoidal salmonellae, impaired gut function might affect drug absorption, justifying the range of doses under study.

 Given this appears primarily to be a dose-finding study for a proposed definitive study, some of the hypotheses, aims and the primary outcomes do not seem to be particularly aligned with this, focusing instead on biomarker and bacterial sepsis endpoints, which the sample size is unlikely to be powered to address effectively. With 35 children per group, and a possible bacterial sepsis rate of 20% only a handful of children in each group are likely to have sepsis. By contrast, the justification for the pharmacokinetic aspects of the study is strong.

 The trial was originally registered in 2017, but logistical delays including COVID-related disruptions led to delayed initiation to January 2021.

 The descriptions of the trial procedures (with a couple of exceptions, noted below) are mostly clear, but there are some points for clarification.

**Major comments:**

1. The co-primary endpoint of “change in CRP at 72 hours” needs better explanation. Malaria itself elevates CRP, so what is the evidence to support using this as a discriminating factor between malaria and malaria+ bacteraemia? There is no discussion to justify this.
2. Control group: 50 children are to be recruited and not treated with azithromycin. At different points these are described as “those hospitalised with severe malaria but not meeting Teule criteria” (which includes severe anaemia, respiratory distress, and prostration, including cerebral malaria, as well as HIV infection); and elsewhere as “non-severe malaria”, Figure 1 and “treatment allocation”, Page 7. Given most severe malaria in Uganda is due to severe anaemia, cerebral malaria and/or respiratory distress and few children who do not meet the Teule criteria but do have severe malaria may be expected, please clarify which children will be in the “control” group.  
3. On page 4 the authors claim that the azithromycin half life is such that it is likely to offer protection from bacteraemia for a month. My reading of the literature is that drug concentrations in target tissues are expected to be above MICs for ~10 days (Lode *et al.*,  J Antimicrob Chemother 1996). ^1^
4. Given dosing is weight based, are there upper and lower weight limits as well as age limits? If so please provide these.
5. Treatment: will the children receive artemether-lumefantrine after they complete parenteral artesunate?
6. Number of blood samples for PK studies: to my reading it was not clear whether this is 4 samples per child (Figure 1)? Or “a total of 7 samples per child” (PKPD substudy, page 10)?
7. Under “statistical analysis” there is no discussion of analysis and interpretation of the PK data which to my mind is the main justification for the study.
8. In the discussion, the authors state “Establishing which children with malaria are at greatest risk of bacteraemia is critical to pragmatically inform a policy for targeted antibiotic therapy that could substantially reduce malaria-associated mortality while minimising the risks of excess antibiotic prescribing.” Whilst true, the present study does not address this question, rather it gives all children in established high risk groups treatment.

**Minor comments:**

1. The methods section of the abstract is hard to follow, particularly the sentence which reads “and investigate azithromycin exposure to change in C-reactive protein, a putative marker of sepsis at 72 hours, and microbiological cure (seven-day) alone and as a composite with seven-day survival.”
2. The list of abbreviations is quite incomplete.
3. In the Introduction, there is a very long sentence that needs revision, starting with “Even when with the best (evidence-based)…”
4. There is no mention of molecular diagnosis of malaria. Will this be performed?
5. Is the LC/MS set up now in Nijmegen? There are published methods for this, using small volumes of plasma.  
6. Under “Trial monitoring” the first sentence is unclear.. It reads: “The Mbale site on to a web enabled trial database will be responsible for its own data entry and local trial management

Is the study design appropriate for the research question?

Yes

Is the rationale for, and objectives of, the study clearly described?

Partly

Are sufficient details of the methods provided to allow replication by others?

Yes

Are the datasets clearly presented in a useable and accessible format?

Not applicable

Reviewer Expertise:

Malaria in pregnancy and childhood. Pathogenesis and immunity. Collaborations with clinical pharmacologists but o specific expertise.

I confirm that I have read this submission and believe that I have an appropriate level of expertise to confirm that it is of an acceptable scientific standard, however I have significant reservations, as outlined above.

## References

[1] : Azithromycin--review of key chemical, pharmacokinetic and microbiological features. *J Antimicrob Chemother* .1996;37 Suppl C: 10.1093/jac/37.suppl_c.1 1-8 10.1093/jac/37.suppl_c.1 8818841

# Response

**Published:** 2022-12-14

**Authors:** Kathryn Maitland

**Affiliations:** KEMRI-Wellcome Trust Research Programme, Kenya

**First of all may I apologise for the late response to Dr Rogerson as we were waiting for a second review so any revisions requested would be included in a single revision. We have still not been able to get an additional review therefore we now provide this response and revised manuscript.** Dr Olupot-Olupot and colleagues present a protocol for a trial that recently commenced recruitment in Uganda, comparing different doses of oral azithromycin as adjunctive treatment for children with severe malaria. Although all doses would be expected to be effective if used parenterally for the commonest pathogen, non-typhoidal salmonellae, impaired gut function might affect drug absorption, justifying the range of doses under study.

 Given this appears primarily to be a dose-finding study for a proposed definitive study, some of the hypotheses, aims and the primary outcomes do not seem to be particularly aligned with this, focusing instead on biomarker and bacterial sepsis endpoints, which the sample size is unlikely to be powered to address effectively. With 35 children per group, and a possible bacterial sepsis rate of 20% only a handful of children in each group are likely to have sepsis. By contrast, the justification for the pharmacokinetic aspects of the study is strong.

 The trial was originally registered in 2017, but logistical delays including COVID-related disruptions led to delayed initiation to January 2021.

 The descriptions of the trial procedures (with a couple of exceptions, noted below) are mostly clear, but there are some points for clarification.

**We thank Dr Rogerson for his very careful consideration of our trial protocol. To be clear the major objectives **are to (i) establish the appropriate dose of oral dispersible azithromycin (by PKPD) as an antimicrobial treatment for children with severe malaria and (ii) investigate whether antibiotics can be targeted to those at greatest risk of bacterial co-infection using clinical criteria alone or in combination with a rapid diagnostic biomarker tests. This study will provide supporting data to inform a future trial and/or policy for hospitals without reliable microbiological facilities. **Here are our specific responses to the comments.**

**Major comments:**

- The co-primary endpoint of “change in CRP at 72 hours” needs better explanation. Malaria itself elevates CRP, so what is the evidence to support using this as a discriminating factor between malaria and malaria+ bacteraemia? There is no discussion to justify this.

The major aim was to see if there were differences in CRP (a putative marker of bacterial sepsis)  between the intervention (Azithromycin) and control. We understand that CRP is elevated in severe malaria, however the data on why CRP is raised is not clear. The most recent review of CRP in malaria only found a handful of studies conducted in Africa, many focused on *P. Vivax* or malaria in pregnancy. (DOI: 10.1038/s41598-021-01556-0). Whilst risk of co-infection could be determined by Teule criteria, this pilot trial will also study a number of other potential biomarkers (which could be done as point of care test) which could identify a group with a higher risk of bacterial sepsis. A continuous numeric (delta change in CRP) endpoint rather than a categorical endpoint reduces the overall number needed. 

- Control group: 50 children are to be recruited and not treated with azithromycin. At different points these are described as “those hospitalised with severe malaria but not meeting Teule criteria” (which includes severe anaemia, respiratory distress, and prostration, including cerebral malaria, as well as HIV infection); and elsewhere as “non-severe malaria”, Figure 1 and “treatment allocation”, Page 7. Given most severe malaria in Uganda is due to severe anaemia, cerebral malaria and/or respiratory distress and few children who do not meet the Teule criteria but do have severe malaria may be expected, please clarify which children will be in the “control” group.  

We apologise for the use of the term non-severe malaria, what was implied was cases admitted with severe malaria who do not have Teule criteria. Those children were identified as the group with the highest risk of bacterial infection. With regards to other cases of severe malaria we include the WHO classification of severe malaria in the PDF file linked here, which considers three groups for admission. Quite a number of other features are not covered by the Tuele critieria.

  

- On page 4 the authors claim that the azithromycin half-life is such that it is likely to offer protection from bacteraemia for a month. My reading of the literature is that drug concentrations in target tissues are expected to be above MICs for ~10 days (Lode *et al.*,  J Antimicrob Chemother 1996). ^1^

We agree the given reference indicates 10 days so we have amended this statement.

  

- Given dosing is weight based, are there upper and lower weight limits as well as age limits? If so please provide these.

We have provided these in the ‘look up table’ with expected upper and lower limits. See Table 1.  

- Treatment: will the children receive artemether-lumefantrine after they complete parenteral artesunate?

Yes, that is correct.

  

- Number of blood samples for PK studies: to my reading it was not clear whether this is 4 samples per child (Figure 1)? Or “a total of 7 samples per child” (PKPD substudy, page 10)?
   There were a total of 7 samples per child. The Figure has “D0, D1-D5 (2 x 0.2ml), D7” where D1-D5 is inclusive of D2, D3 and D4.

Under Figure 1 where we state D0, D1- (2x0.2ml*), D7 the asterix was missing a label, which now indicates D1-D5 inclusive.

  

- Under “statistical analysis” there is no discussion of analysis and interpretation of the PK data which to my mind is the main justification for the study.
- Of methods used for the PK analysis is described under the PKPD substudy section of the paper. More details will be given in the trial report since the model will need to be built on the clinical data.
- In the discussion, the authors state “Establishing which children with malaria are at greatest risk of bacteraemia is critical to pragmatically inform a policy for targeted antibiotic therapy that could substantially reduce malaria-associated mortality while minimising the risks of excess antibiotic prescribing.” Whilst true, the present study does not address this question, rather it gives all children in established high risk groups treatment.

We agree; this is a pilot study aimed at informing a future trial with those aims.

**Minor comments:**

- The methods section of the abstract is hard to follow, particularly the sentence which reads “and investigate azithromycin exposure to change in C-reactive protein, a putative marker of sepsis at 72 hours, and microbiological cure (seven-day) alone and as a composite with seven-day survival.”
- has been revised to ‘and investigate the impact of different levels of azithromycin exposure on change in C-reactive protein (a putative marker of sepsis at 72 hours), as well as on microbiological cure alone and as a composite with survival at 7 days’.
- The list of abbreviations is quite incomplete.

We have updated these.

  

- In the Introduction, there is a very long sentence that needs revision, starting with “Even when with the best (evidence-based)…”

This has been revised.

  

- There is no mention of molecular diagnosis of malaria. Will this be performed?

There is no need to perform molecular diagnosis of malaria – this is a clinical study where children will be enrolled pragmatically using clinical criteria, point of care malaria diagnostics and a malaria slide. We are also performing PfHRP2 on admission samples to characterise those with true severe malaria from those with incidental parasitaemia.

  

- Is the LC/MS set up now in Nijmegen? There are published methods for this, using small volumes of plasma.  

 This is currently being set up in Nijmegen; the methods will be published separately.

- Under “Trial monitoring” the first sentence is unclear.. It reads: “The Mbale site on to a web enabled trial database will be responsible for its own data entry and local trial management

  We have corrected this sentence to provide more clarity.
